# Decreased Lipid Phosphate Phosphatase 1/3 and Increased Lipid Phosphate Phosphatase 2 Expression in the Human Breast Cancer Tumor Microenvironment Promotes Tumor Progression and Immune System Evasion

**DOI:** 10.3390/cancers15082299

**Published:** 2023-04-14

**Authors:** Matthew G. K. Benesch, Rongrong Wu, Xiaoyun Tang, David N. Brindley, Takashi Ishikawa, Kazuaki Takabe

**Affiliations:** 1 Department of Surgical Oncology, Roswell Park Comprehensive Cancer Center, Buffalo, NY 14263, USA; matthew.benesch@roswellpark.org; 2 Department of Breast Surgery and Oncology, Tokyo Medical University, Tokyo 160-8402, Japan; rongrong.wu@roswellpark.org (R.W.); tishik55@gmail.com (T.I.); 3 Cancer Research Institute of Northern Alberta, Department of Biochemistry, University of Alberta, Edmonton, AB T6G 2H7, Canada; xtang2@ualberta.ca (X.T.); david.brindley@ualberta.ca (D.N.B.); 4 Department of Gastroenterological Surgery, Yokohama City University Graduate School of Medicine, Yokohama 236-0004, Japan; 5 Division of Digestive and General Surgery, Niigata University Graduate School of Medical and Dental Sciences, Niigata 951-8520, Japan; 6 Department of Breast Surgery, Fukushima Medical University School of Medicine, Fukushima 960-1295, Japan; 7 Department of Surgery, University at Buffalo Jacobs School of Medicine and Biomedical Sciences, State University of New York, Buffalo, NY 14263, USA

**Keywords:** bioinformatics, cell cycle, lysophosphatidic acid, novel therapeutics, tumor progression, signal transduction

## Abstract

**Simple Summary:**

The lipid phosphate phosphatases (LPPs) are a family of three enzymes that act at the cell surface and within the cell to modulate signaling. In many tumors, including breast cancers, LPP1 and LPP3 expression levels are decreased and LPP2 expression levels are increased relative to normal tissue, which stimulates a pro-cancerous phenotype by decreasing the turnover of bioactive lipids and promoting cell cycle progression. While most research has been conducted in the laboratory, validation in human tumors is lacking. We used three large independent patient cohorts and single-cell RNA-sequencing data to show that decreased LPP1/3 and increased LPP2 expression in cancers correlated to worse tumor biology, immune system evasion, and decreased survival. Most tumor LPP1/3 is produced by the stroma and LPP2 by cancer cells. Overall, our findings support pre-clinical evidence that restoring tumor LPP expression balance, particularly through LPP2 inhibition, could provide adjunct therapies for breast cancer patients.

**Abstract:**

The LPP family is comprised of three enzymes that dephosphorylate bioactive lipid phosphates both intracellularly and extracellularly. Pre-clinical breast cancer models have demonstrated that decreased LPP1/3 with increased LPP2 expression correlates to tumorigenesis. This though has not been well verified in human specimens. In this study, we correlate LPP expression data to clinical outcomes in over 5000 breast cancers from three independent cohorts (TCGA, METABRIC, and GSE96058), investigate biological function using gene set enrichment analysis (GSEA) and the xCell cell-type enrichment analysis, and confirm sources of LPP production in the tumor microenvironment (TME) using single-cell RNA-sequencing (scRNAseq) data. Decreased LPP1/3 and increased LPP2 expression correlated to increased tumor grade, proliferation, and tumor mutational burden (all *p* < 0.001), as well as worse overall survival (hazard ratios 1.3–1.5). Further, cytolytic activity was decreased, consistent with immune system invasion. GSEA data demonstrated multiple increased inflammatory signaling, survival, stemness, and cell signaling pathways with this phenotype across all three cohorts. scRNAseq and the xCell algorithm demonstrated that most tumor LPP1/3 was expressed by endothelial cells and tumor-associated fibroblasts and LPP2 by cancer cells (all *p* < 0.01). Restoring the balance in LPP expression levels, particularly through LPP2 inhibition, could represent novel adjuvant therapeutic options in breast cancer treatment.

## 1. Introduction

Despite excellent prognosis if detected and treated as a local disease, between 3 and 15% of breast cancers will relapse within 10 years, and up to 36% of patients will initially present with either nodal or metastatic disease [1,2]. In the United States alone, treatment resistance and disease progression account for over 40,000 breast cancer deaths annually [3,4]. This ultimate bastion of treatment disease relapse and treatment failure is a fundamental target of ongoing breast cancer research [5].

**Figure 1:**
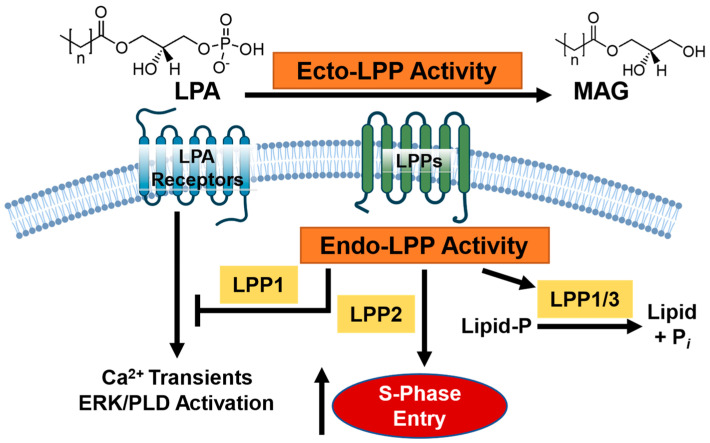
Overview of the current model of lipid phosphate phosphatase (LPP) signaling in breast cancer. The LPPs comprise a family of three enzymes, capable of dephosphorylating extracellular lipid phosphates at the cell membrane surface (ecto-LPP activity) such as lysophosphatidate (LPA) into monoacylglycerol (MAG). LPPs can regulate substrates inside the cell through either enzymatic or catalytic activities (endo-LPP activity), such as via lipid dephosphorylation; promotion of S-phase cell entry (LPP2 specifically); or modulation of downstream calcium transient, ERK, and phospholipase D (PLD) activation. Broadly speaking, decreased LPP1/3 activity and increased LPP2 activity is associated with a pro-cancer phenotype.

The lipid phosphate phosphatases (LPPs) are an increasingly attractive and novel target for adjuvant therapy development [6,7]. The LPPs are a family of three enzymes (LPP1–3) that function at the plasma membrane and internal membranes of organoids such as the endoplasmic reticulum and the Golgi apparatus [8,9,10]. At the plasma membrane, the catalytic sites of the LPPs face the extracellular side (ecto-activity) and function to dephosphorylate multiple bioactive lipids, including extracellular sphingosine 1-phosphate (S1P) and particularly lysophosphatidate (LPA) (Figure 1) [11]. LPA is produced from lysophosphatidylcholine (LPC), primarily by the lysophospholipase D activity of the secreted enzyme autotaxin (ATX) [12,13]. LPA signals through six membrane-bound G-protein coupled receptors to facilitate tumor proliferation, metastasis, and therapy resistance in cancer cells [6,14]. To favor tumorigenesis, we have shown in murine models that tumors increase LPA concentrations by overexpressing ATX themselves, or by cytokine stimulation in the tumor microenvironment (TME) stroma, and decrease the expression of LPP1 and LPP3 [4]. In breast and ovarian murine allograft and xenograft cancer models, increasing these low levels of either LPP1 or LPP3 in implanted cancer cells slows subsequent tumor growth and metastasis [15,16]. We previously showed that low LPP1 expression in human MDA-MB-231 and murine Balb/c 4T1 breast cancer cells increases levels of cyclin D1 and D3 and matrix metalloproteinases via transcription of multiple factors, which ultimately leads to cell division [17]. Conversely, LPP2 expression is upregulated in many cancers, including breast, with its catalytic activity inside the cells (endo-LPP functions) (Figure 1) [7,8]. These functions increase S-phase entry in the cell cycle via upregulation of the c-Myc transcription factor [18,19,20]. We have additionally shown that LPP2 knockout in MDA-MB-231 breast cancer cells slows tumor growth and lung micro-metastasis in a xenograft mouse model [20]. The cell biology of the LPPs is further reviewed elsewhere [8,15,17,20,21,22,23,24,25,26].

Pre-clinical investigations in both cell cultures and murine tumor models have provided considerable support for inhibitors against the ATX-LPAR-LPP pathway, some of which, especially against ATX and the LPARs, are currently in clinical trials [4,27,28,29,30,31]. However, there are no therapies under clinical investigation to restore LPP1/3 levels or suppress LPP2 levels in cancers. There are limited studies to validate LPP investigative findings in human tumors and, particularly, the role of LPPs in the cancer TME is largely unknown. In this study, we explore the role of LPP mRNA expression (LPP1 gene name *PLPP1* and historically *PAPP2A*; LPP2 gene name *PLPP2*, *PAPP2C*; LPP3 gene name *PLPP3*, *PAPP2B*) [8] within the human breast cancer TME via in silico research approaches using three large independent cohorts. These results should assist in making meaningful comparative analyses to extensive pre-clinical investigations that support the potential of future pharmacological development to mitigate the pathological balance of LPP expression in tumors.

## 2. Materials and Methods

### 2.1. Data Acquisition

Clinical and mRNA expression breast cancer data were obtained from the Cancer Genome Atlas Program (TCGA) (whole database, *n* = 1090; estrogen receptor positive and human epidermal growth factor receptor negative tumors (ER+ HER2−), *n* = 593; human epidermal growth factor receptor positive tumors (HER2+), *n* = 184; and triple negative breast cancer (TNBC), *n* = 160), the Molecular Taxonomy of Breast Cancer International Consortium (METABRIC) (whole database, *n* = 1094; ER+ HER2−, *n* = 1355; HER2+, *n* = 236; and TNBC, *n* = 313), and GSE96058 (whole database, *n* = 3069; ER+ HER2−, *n* = 2277; HER2+, *n* = 392; and TNBC, *n* = 155). These three databases were obtained via the cBioPortal (https://www.cbioportal.org (accessed on 9 October 2022)) and the Gene Expression Omnibus (GEO) repository of the United States National Institutes of Health (https://www.ncbi.nlm.nih.gov/geo (accessed on 9 October 2022)), as previously described [32,33]. Gene expression data from 114 samples of normal breast tissue were obtained from the Genotype-Tissue Expression (GTex) Portal (https://gtexportal.org (accessed on 9 October 2022)) [34]. Single-cell RNA sequencing breast cancer atlas data were also acquired for LPP expression from two large published studies [35,36] via the Broad Institute Single Cell Portal (https://singlecell.broadinstitute.org/single_cell (accessed on 9 October 2022)). As all data were obtained from deidentified public resources, ethics approval requirements were waived by the Roswell Park Institutional Review Board.

### 2.2. Gene Set Enrichment Analysis

Functional enrichment analysis of the LPP genes (*PLPP1–3*) was performed by gene set enrichment analysis (GSEA) [33] on the Molecular Signatures Database Hallmark collection (http://www.gsea-msigdb.org (accessed on 9 October 2022)) [37]. A false discovery rate (FDR) of <0.25 indicated enriched signaling gene sets [38]. The cohorts were dichotomized into high and low LPP expression groups by median gene expression. A positive NES score signifies enriched signaling in the LPP-high expression group and a negative NES score signifies enriched signaling in the LPP-low expression group.

### 2.3. Other Scores

The xCell algorithm (https://xcell.ucsf.edu (accessed on 9 October 2022)) [39] was used to correlate LPP gene expression to the infiltrating fraction of TME stromal cells (adipocytes, preadipocytes, fibroblasts, endothelial cells, and pericytes) and immune cells (CD8+, T helper cell (Th)1 and Th2 cells, T-regulator cells, M1 and M2 macrophages, and dendritic cells), as previously described [40,41,42,43]. The breast cancer mutational landscape (intratumor heterogeneity, homologous recombination defects, fraction genome altered, silent mutation rate, non-silent mutation rate, single-nucleotide neoantigens, and indel mutations), proliferation score, stromal fraction, TGF-β score, and immune scores (leukocyte fraction, lymphocyte infiltration, tumor infiltration lymphocyte fraction, macrophage regulation, and wound healing) were derived from Thorsson et al. [44]. TME immune cytolytic activity (CYT) was calculated with the xCell algorithm as the geometric mean of the expression of perforin (*PRF1*) and granzyme A (*GZMA*) mRNA expression, which are measures of cytotoxic T cell anti-cancer abilities [45].

### 2.4. Statistical Anlayses

Statistical analyses were conducted with R 4.2.1 (https://www.R-project.org (accessed on 9 October 2022)). Graphics were produced with the R software package and Origin Pro 2022 (OriginLab Corporation, Northampton, MA, USA). LPP mRNA levels were dichotomized into low and high groups based on the median expression level. All results are plotted as box plots, with the lower and upper bounds representing the maximum and minimum values; the upper and lower ends of the box representing the 25th and 75th percentile values, respectively; and the bolded bar within the box representing the median value. Multiple group comparisons were performed using the Kruskal–Wallis test and two-group comparisons were performed using the Wilcoxon signed-rank test. The R survival software package was used to analyze disease-free survival (DFS), disease-specific survival (DSS), and overall survival (OS) based on high or low LPP expression via Cox proportional hazards regression. *p* < 0.05 was set for statistical significance.

## 3. Results

### 3.1. High LPP1 and LPP3 Gene Expression and Low LPP2 Gene Expression Correlate to a Less Aggressive Breast Cancer Phenotype

**Figure 2:**
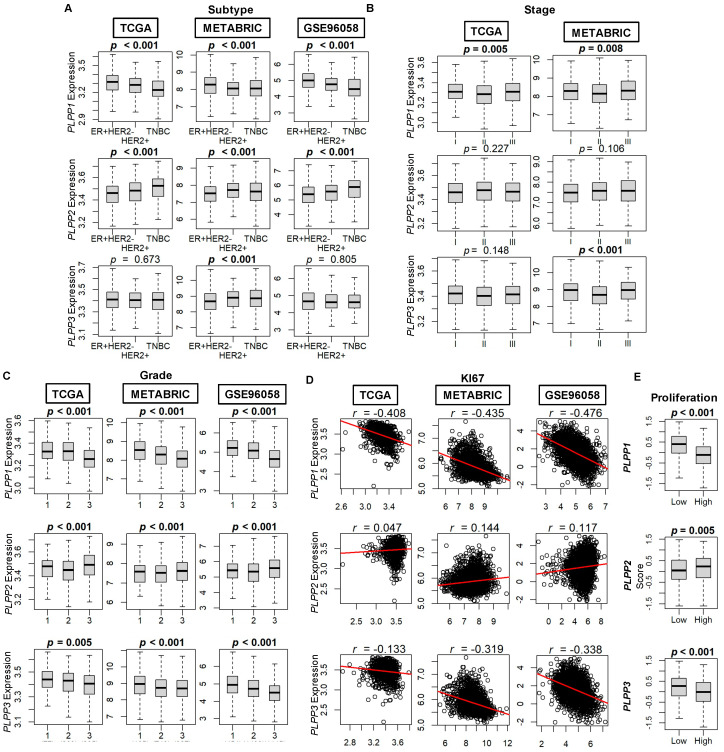
LPP gene expression by breast cancer tumor subtype, stage, grade, and proliferation. (**A**) Breast cancer subtype. ER+ HER2− (estrogen receptor positive, human epidermal growth factor receptor negative tumors), HER2+, and TNBC (triple negative breast cancer). Counts by cohort: TCGA (ER+ HER2− *n* = 593, HER2+ *n* = 184, TNBC *n* = 160), METABRIC (ER+ HER2− *n* = 1355, HER2+ *n* = 236, TNBC *n* = 313), and GSE96058 (ER+ HER2− *n* = 2277, HER2+ *n* = 392, TNBC *n* = 155). (**B**) Staging according to the American Joint Committee on Cancer (AJCC). Stage is not available for the GSE96058 cohort. Counts by cohort: TCGA (stage I *n* = 181, stage II *n* = 617, stage III *n* = 248) and METABRIC (stage I *n* = 475, stage II *n* = 800, stage III *n* = 115). (**C**) Breast cancer grade. Counts by cohort: TCGA (grade 1 *n* = 77, grade 2 *n* = 269, grade 3 *n* = 235), METABRIC (grade 1 *n* = 165, grade 2 *n* = 740, grade 3 *n* = 927), and GSE96058 (grade 1 *n* = 454, grade 2 *n* = 1439, grade 3 *n* = 1115). (**D**) Ki67 correlation plots to LPP expression. (**E**) Proliferation score. Database on the scores by Thorsson, et al. [44]. Multiple group comparisons were performed using the Kruskal–Wallis test, and two-group comparisons were performed using the Wilcoxon signed-rank test. The bolded center bar within the box plots represents the median; the lower and upper box bounds represent the 25th and 75th percentiles, respectively; and the lower and upper tails represent the minimum and maximum values, respectively.

We correlated the expression of LPP1–3 to characteristics of the breast tumors in each of the three cohorts. In all three cohorts, LPP1 (*PLPP1*) gene expression is lowest for TNBC tumors and highest for ER+ HER2− tumors (all *p* < 0.001, Figure 2A). This trend is essentially reversed for LPP2 (*PLPP2*) gene expression (all *p* < 0.001), whereas there is no difference across the three subtypes based on LPP3 (*PLPP3*) gene expression (Figure 2A). Data for disease stage are available from TCGA and METABRIC cohorts, and the only consistent result between the two cohorts is that median LPP1 gene expression is lowest for stage II tumors compared with stage I and III tumors (*p* < 0.001, Figure 2B). When analyzed by tumor grade, both LPP1 and LPP3 gene expression decreased monotonically with increasing grade in all three cohorts (all *p* < 0.001, Figure 2C). Median LPP2 expression was slightly lower for grade II tumors than for grade I tumors, but the highest expression occurred in grade III tumors in all cohorts (all *p* < 0.001, Figure 2C). Ki67 expression, a marker of cell proliferation, was negatively correlated with increasing LPP1/3 expression (Figure 2D) and, likewise, proliferation scores by Thorsson, et al. [44] were significantly decreased for high LPP1- and LPP3-expressing tumors compared with low expressing tumors (*p* < 0.001, Figure 2E). The reverse trend, however, occurred for LPP2 when comparing high and low expressing tumors and Ki67 correlation (*p* = 0.005, Figure 2D,E).

When dichotomized by nodal status (negative or positive), there was no correlation to high or low LPP gene expression (Figure S1A). There are only 20 samples from metastatic tumors in TCGA and 9 samples in METABRIC. Therefore, reliable conclusions cannot be made between LPP gene expression and the presence of metastasis (Figure S1B).

**Figure 3:**
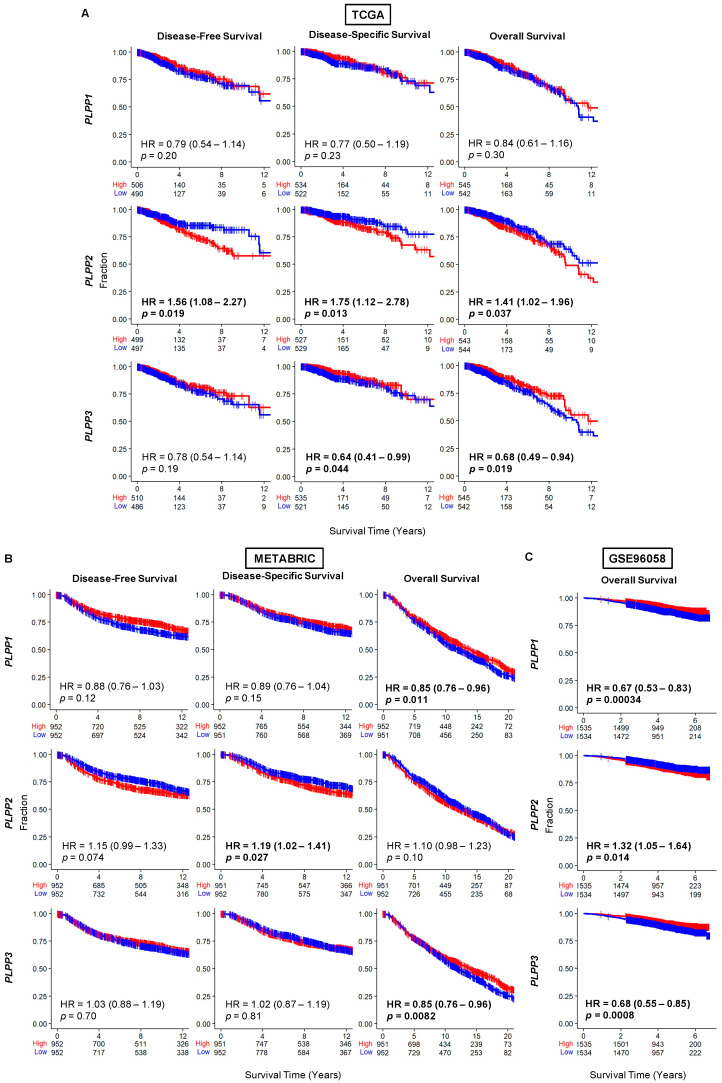
Survival plots for low and high LPP gene expression in breast cancer tumors for the whole cohort for each dataset. (**A**) TCGA cohort results. (**B**) METABRIC cohort results. (**C**) GSE96058 cohort results. Patients at risk for each time point are listed along the *x*-axis. LPP gene expression is dichotomized into low and high groups by the median. The hazard ratio (HR) compares the high group against the low group.

We next studied survival trends based on the median dichotomization of LPP gene expression. The results are expressed as disease-free survival (DFS), disease-specific survival (DSS), and overall survival (OS). Upon examination of the whole cohort, DFS and DSS favored high LPP1 gene expression but did not reach significance (Figure 3). However, OS was significantly better in both the METABRIC and GSE96058 cohorts, and trended towards significance for the high LPP1 gene expression patients (hazard ratio (HR) estimates about 0.63–0.85, Figure 3). A near identical pattern occurred in LPP3 gene expression, but overall survival was significantly better across all three cohorts for high LPP3-expressing tumors compared with low expression tumors (HR 0.68–0.85, all *p* < 0.02, Figure 3). In the TCGA cohort, high LPP2 expression correlated significantly with reduced DFS, DSS, and OS (HRs 1.4–1.7, all *p* < 0.02, Figure 3A). This same trend occurred in the METABRIC cohort, but it was only statistically significant for DSS (HR 1.19 (1.02–1.41), *p* = 0.03, Figure 3B). OS was significantly decreased for high LPP2-expressing tumors compared with low LPP2-expressing tumors in the GSE96058 cohort (HR 1.32 (1.05–1.64), *p* = 0.01, Figure 3C). The results were then sub-analyzed by hormone status in Figures S2–S4.

**Figure 4:**
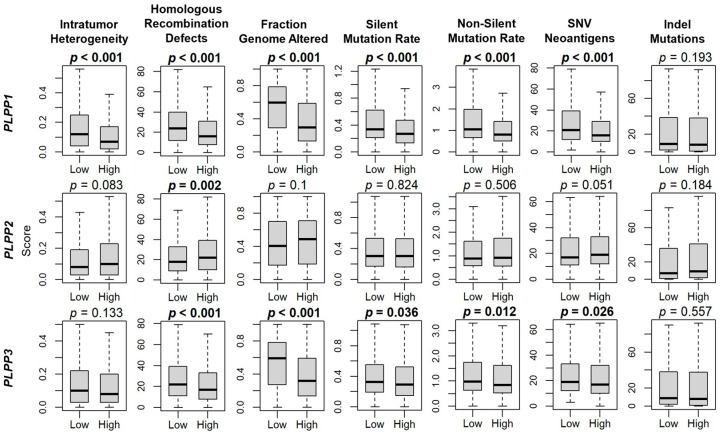
LPP gene expression correlation with breast cancer mutations. Box plots of intratumor heterogeneity, homologous recombination defects, fraction genome altered, silent mutation rate, non-silent mutation rate, single-nucleotide variant (SNV) neoantigens, and indel mutations. Data are based on the scores by Thorsson, et al. [44]. LPP gene expression is dichotomized into low and high groups by the median. The bolded center bar represents the median; the lower and upper box bounds represent the 25th and 75th percentiles, respectively; and the lower and upper tails represent the minimum and maximum values, respectively.

Because tumor mutational burden can often be a marker of cancer aggressiveness in many tumors [42], we next examined its correlation with LPP gene expression. Intratumor heterogeneity was significantly decreased for high LPP1-expressing tumors (*p* < 0.001), but though not significant, trended lower for high LPP3-expressing tumors, and trended higher for high LPP2-expressing tumors (Figure 4). This decreased pattern for high LPP1- and LPP3-expressing tumors, and increased level for high LPP2-expressing tumors, was statistically significant for homologous recombination defects (all *p* ≤ 0.002, Figure 4). Fraction genome altered, silent mutation rate, non-silent mutation rate, and single-nucleotide variant neoantigens were all significantly decreased with high LPP1 and LPP3 expression (all *p* < 0.001), but they were not different for LPP2 (Figure 4). There were no significant differences in indel mutations for any of the LPPs (Figure 4).

### 3.2. LPP1- and LPP3-Mediated Gene Set Enrichment Patterns Favor a Mixed Pro- and Anti-Cancer Phenotype, whereas LPP2 Patterns Favor Cell Cycling Progression Pathways

**Figure 5:**
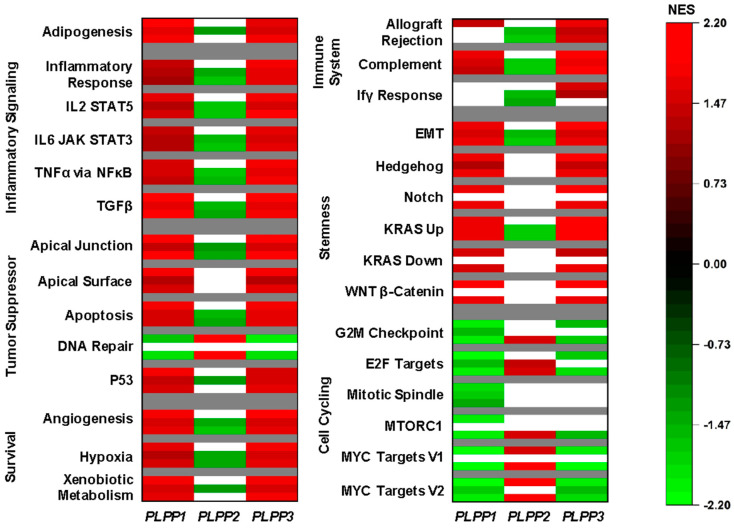
Gene set enrichment analysis (GSEA) for the LPPs in breast cancer. For all Hallmark gene sets listed, the top bar indicates the normalized enrichment score (NES) from the TCGA cohort, the middle bar from the METABRIC cohort, and the lower bar from the GSE96508 cohort. A false discovery rate (FDR) of less than 0.25 was considered statistically significant. White bars indicate no significance.

We used gene set enrichment analysis (GSEA) on Hallmark pathways to study correlations to LPP gene expression [37]. Gene sets were selected if they had significance in at least two cohorts. The complete GSEA output is presented in Table S1. LPP1 and LPP3 shared similar patterns. Specifically, the most consistently upregulated gene sets in high LPP1- and LPP3-expressing tumors were related to inflammatory signaling and survival pathways (Figure 5). Additionally, the adipogenesis gene set was significantly upregulated in all three cohorts, suggesting that high LPP1 and LPP3 expression correlated to stromal cell populations. The most upregulated gene sets in high LPP2-expressing tumors were cell cycling gene sets, particularly the E2F and MYC targets (Figure 5).

### 3.3. LPP1 and LPP3 Are Predominantly Expressed in Tumor Stroma, While LPP2 Is Expressed in Cancer Cells, and a Low Tumor LPP1/3 and High LPP2 Expression Pattern Correlates to Immune System Evasion

**Figure 6:**
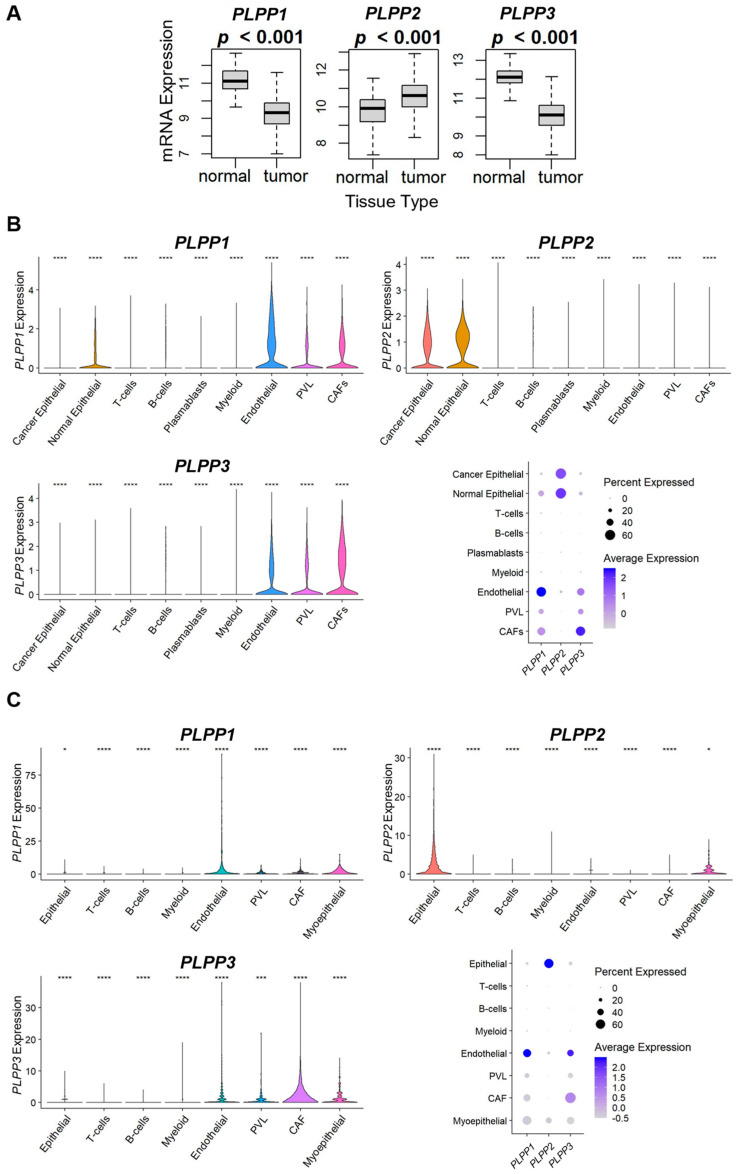
LPP gene expression in normal tissues and tumors and violin and dot plot analysis of LPP expression for single cell RNA sequencing of breast cancer tumors. (**A**) LPP gene expression from 114 normal breast tissues is compared to 1090 breast cancer tumors from the TCGA database. The results are plotted as box plots. The bolded center bar represents the median; the lower and upper box bounds represent the 25th and 75th percentiles, respectively; and the lower and upper tails represent the minimum and maximum values, respectively. (**B**) Single-cell RNA sequencing results from the cohort described in [35], comprised of 26 tumors (11 ER+ HER2−, 5 HER2+, and 10 TNBC), with a total of 130,246 single cells. (**C**) Single-cell RNA sequencing results from the cohort described in [36], comprised of 5 TNBC tumors, with a total of 24,271 single cells. For results in (**B**,**C**), violin plot results for the individual LPP genes are shown following *t*-test for each type compared with the base mean (* *p* ≤ 0.05, *** *p* ≤ 0.001, **** *p* ≤ 0.0001). The summary chart shows the overall percentage of the total LPP expression by cell type and the average expression within each cell type for each cohort.

We next examined LPP expression within the tumor microenvironment. When comparing whole breast tumors to normal breast tissues, tumors expressed significantly less LPP1 and LPP3 than normal tissue, while tumors expressed more LPP2 than normal tissues (Figure 6A). We then examined LPP gene expression in two cohorts of single-cell RNA sequencing to determine the cell origins of predominant expression. Tumor LPP1 was primarily expressed in endothelial cells, followed by cancer-associated fibroblasts (CAFs), whereas most LPP3 was associated with CAFs, followed by endothelial cells (Figure 6B,C). For epithelial cells, LPP1/3 expression was decreased in cancer epithelial cells compared with normal epithelial cells (Figure 6B,C). Virtually all LPP2 expression was epithelial-cell-based (cancer and normal), followed by myoepithelial cells (Figure 6B,C).

**Figure 7:**
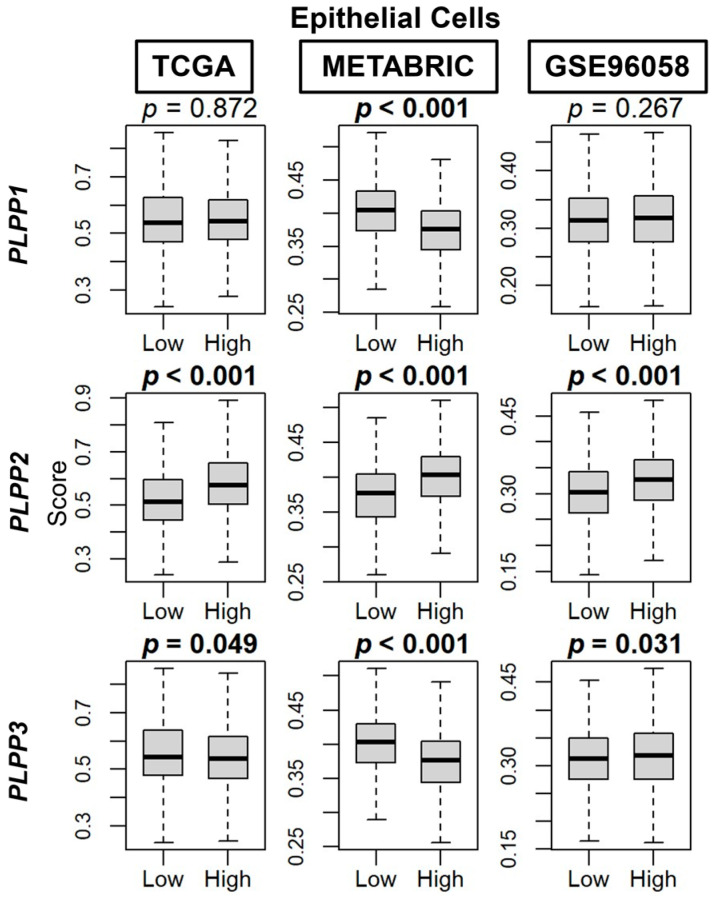
Epithelial cell composition correlation with LPP expression in breast cancer tumors. Box plots are based on the xCell algorithm for the TCGA, METABRIC, and GSE96058 cohorts. LPP gene expression is dichotomized into low and high groups by the median. The bolded center bar represents the median; the lower and upper box bounds represent the 25th and 75th percentiles, respectively; and the lower and upper tails represent the minimum and maximum values, respectively.

We then used the xCell algorithm to examine tumor cell populations based on dichotomized LPP expression. Epithelial cell (primarily cancer cells) composition was significantly enriched in high LPP2 tumors across all three cohorts (*p* < 0.001, Figure 7). For LPP1 and LPP3, there was no significant trend or correlation to epithelial cell composition (Figure 7).

**Figure 8:**
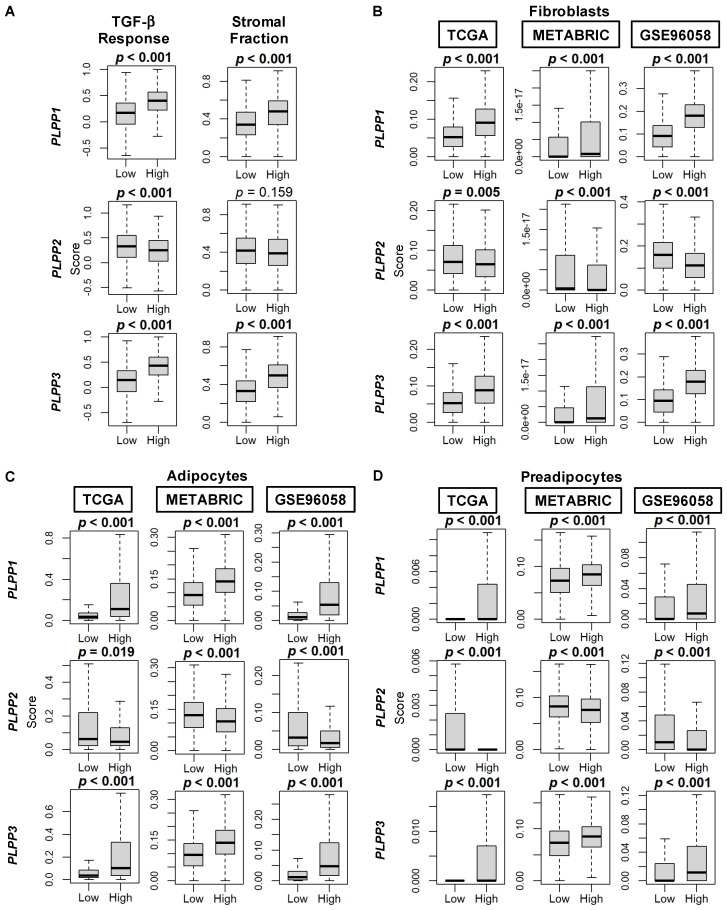
TGF-β and stromal fraction scores, and fibroblast, adipocyte, and preadipocyte composition correlation with LPP gene expression in breast cancer tumors. (**A**) Box plots of TGF-β and stromal fraction scores. Data are based on the scores by Thorsson, et al. [44]. (**B**) Box plots of fibroblast cell composition. (**C**) Box plots of adipocyte cell composition. (**D**) Box plots of preadipocyte cell composition. Data in **B**–**D** based on the xCell algorithm for the TCGA, METABRIC, and GSE96058 cohorts. LPP gene expression is dichotomized into low and high groups by the median. The bolded center bar represents the median; the lower and upper box bounds represent the 25th and 75th percentiles, respectively; and the lower and upper tails represent the minimum and maximum values, respectively.

**Figure 9:**
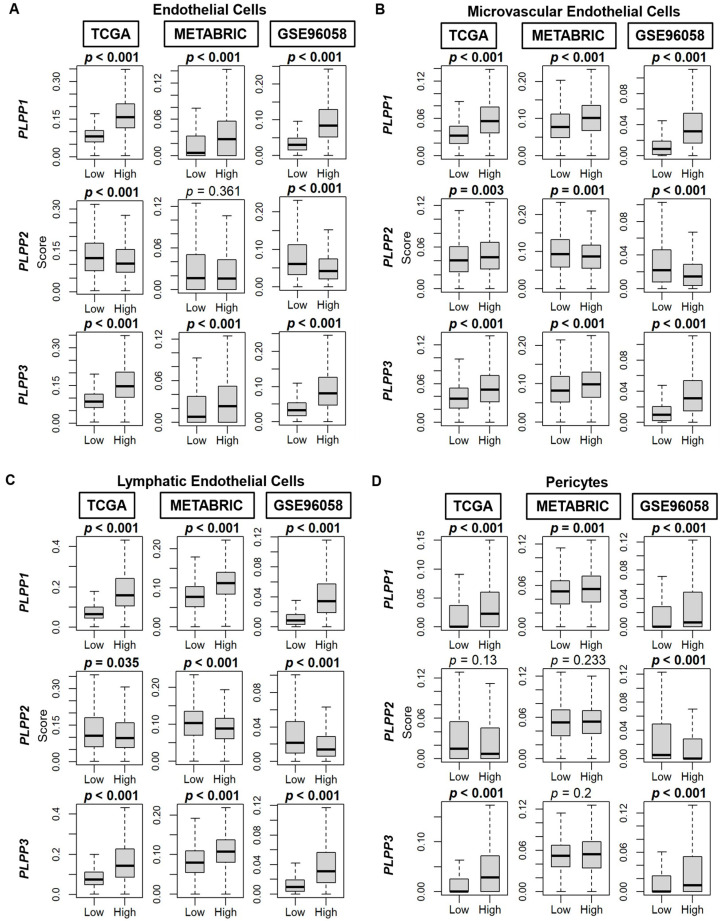
Endothelial cell, microvascular endothelial cell, lymphatic endothelial cell, and pericyte composition correlation with LPP gene expression in breast cancer tumors. (**A**) Box plots of endothelial cell composition. (**B**) Box plots of microvascular endothelial cell composition. (**C**) Box plots of lymphatic endothelial cell composition. (**D**) Box plots of pericyte composition. All data are based on the xCell algorithm for the TCGA, METABRIC, and GSE96058 cohorts. LPP gene expression is dichotomized into low and high groups by the median. The bolded center bar represents the median; the lower and upper box bounds represent the 25th and 75th percentiles, respectively; and the lower and upper tails represent the minimum and maximum values, respectively.

Scores by Thorsson et al. [44] demonstrated an enriched TGF-β response, a marker of stromal fibrosis, in high LPP1- and LPP3-expressing tumors, as well as decreased scores in high LPP2-expressing tumors (all *p* < 0.001, Figure 8A). These patterns of high cell type composition in high LPP1- and LPP3-expressing tumors and low cell type composition in high LPP2-expressing tumors were subsequently significantly reflected in the xCell algorithm scores of TME fibroblasts, adipocytes, and preadipocytes in all three cohorts (all *p* < 0.001, Figure 8B–D). Similarly, overall endothelial cell composition was significantly enriched for both high LPP1- and LPP3-expressing tumors, and additionally for both microvascular and lymphatic endothelial cells (all *p* < 0.001, Figure 9A–C). The results for LPP2 were more variable. Endothelial cell composition was significantly reduced in high LPP2-expressing tumors in TCGA and GSE96058 (*p* < 0.001), but not in METABRIC (Figure 9A). Microvascular endothelial cell composition was slightly increased in TCGA cohort in high LPP2-expressing tumors, but decreased in the other two cohorts (all *p* < 0.005, Figure 9B). In all three cohorts, lymphatic endothelial cell composition was significantly decreased in high LPP2-expressing tumors (Figure 9C). In high LPP1-expressing tumors, pericyte composition was significantly increased in all three cohorts (all *p* ≤ 0.001, Figure 9D), but there were no consistently significant results for the other two LPPs (Figure 9D).

Immune cell populations were then correlated to LPP gene expression. For anti-cancer CD8+ T cells, LPP1 and LPP2 did not demonstrate significance or a consistent trend across the three cohorts, but for the high LPP3-expressing tumors, CD8+ T cells were slightly elevated compared with the low LPP3-expressing tumors (all *p* < 0.001, Figure S5A). Th1 cells were decreased in the high LPP1- and LPP3-expressing tumors, but increased for high LPP2-expressing tumors (all *p* < 0.001, Figure S5B). M1 macrophages were decreased in the high LPP1-expressing tumors (all *p* < 0.001), but there was no effect across the cohorts for LPP2 and LPP3 (Figure S5C). Dendritic cells were significantly increased in both high LPP1- and LPP3-expressing tumors across all three cohorts (all *p* < 0.02), and decreased for high LPP2-expressing tumors in the METABRIC and GSE96058 cohorts (*p* < 0.001, Figure S5D). Among the pro-cancer immune cell populations, Tregs were significantly reduced in high LPP1-expressing tumors across all three cohorts (*p* < 0.001), but no consistent trends were seen for LPP2 or LPP3 (Figure S6A). For Th2 cells, there was no concordance in results across the three cohorts for any of the LPPs (Figure S6B). High LPP2-expressing tumors had lower levels of M2 macrophages (all *p* < 0.02), but there were no consistent differences for LPP1/3 tumors (Figure S6C).

**Figure 10:**
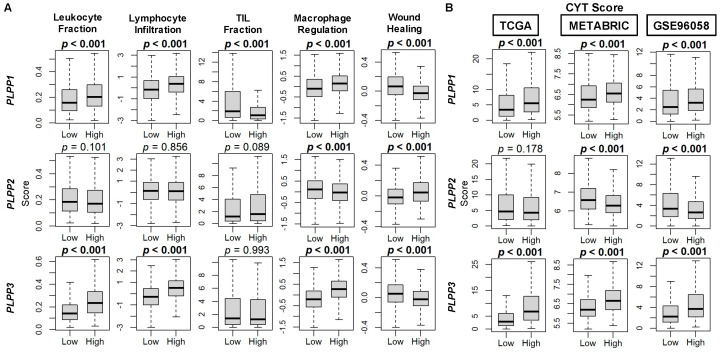
Immune scores for markers of tumor immune cell populations and cytolytic (CYT) score correlation with LPP gene expression in breast cancer tumors. (**A**) Box plots of immune score results are based on score by Thorsson, et al. [44]. (**B**) Box plots of CYT scores based on the xCell algorithm for TCGA, METABRIC, and GSE96058 cohorts. LPP gene expression is dichotomized into low and high groups by the median. The bolded center bar represents the median; the lower and upper box bounds represent the 25th and 75th percentiles, respectively; and the lower and upper tails represent the minimum and maximum values, respectively.

Taken together, upon examination of immune-related scores by Thorsson, et al. [44], high LPP1- and LPP3-expressing tumors had higher scores for leukocyte fraction, lymphocyte infiltration, and macrophage regulation than their low expressing counterparts (all *p* < 0.001, Figure 10A). For LPP2 tumors, among these three scores, macrophage regulation scores were decreased in high LPP2-expessing tumors (*p* < 0.001, Figure 10A). The tumor-infiltrating lymphocyte (TIL) fraction was decreased in high LPP1-expressing tumors (*p* < 0.001), but there was no difference by LPP2 or LPP3 expression (Figure 10A). The wound healing score was decreased in both high LPP1- and LPP3-expressing tumors and increased in high LPP2-expressing tumors (all *p* < 0.001, Figure 10B). Finally, upon analysis of cytolytic activity (CYT), both high LPP1- and LPP3-expressing tumors had significantly higher CYT scores compared with low expressing tumors in all three cohorts (all *p* < 0.001), while high LPP2-expressing tumors had lower CYT scores only in the METABRIC and GSE96058 cohorts (both *p* < 0.001, Figure 10B).

## 4. Discussion

Pathological expression of LPPs in cancers, including breast cancer, is canonically characterized by decreased LPP1/3 and increased LPP2 expression. This finding has been well documented in multiple lines of investigation using cancer cell cultures and extended to murine allograft and xenograft orthotopic breast cancer models [8,9,15,17,19,20]. As a result, particularly attributable to decreased LPP1/3, turnover of extracellular LPA and other bioactive lipids is decreased in breast cancer cell cultures and tumors relative to normal breast tissue. When coupled to increased LPA production through increased tumor microenvironment ATX expression and activity, the net effect is an increase in LPA concentrations for signaling through LPARs to amplify disease progression [4,6]. We previously showed that breast cancer cells transduced with catalytically active LPP1 form smaller tumors with decreased metastasis in orthotopic mouse models [15]. Mechanistically, low LPP1 expression is associated with increased levels of cyclin D1/D3 and matrix metalloproteinases secondary to increased transcription by cFOS and cJUN [17]. Similar work in SKOV3 ovarian cancer cells also demonstrated decreased tumor growth in nude mice with LPP3 overexpression [46]. While LPP1 and LPP3 may function in a seemingly complementary manner, there are important physiological differences between the two enzymes: LPP3 knockout mice are not viable [47], whereas both LPP1- and LPP2-knockout mice are viable [26,48]. The effects of LPP2 on tumor growth are significantly different from LPP1/3, in that its overexpression in cancer cells leads to premature S-phase cell entry via upregulation of c-Myc nuclear expression [19,20].

The findings of this study agree with the LPP cancer cell biology of pre-clinical investigations [8,9,15,17,19,20] and represent the most comprehensive analysis to date in human breast tumors, with over 5000 specimens examined in three independent databases. We confirmed on whole tumor analysis that breast tumors had lower LPP1/3 and higher LPP2 expression compared with normal tissue. On single-cell RNA-seq analysis, most tumor LPP1/3 was expressed in tumor stromal cell populations, such as endothelial cells and fibroblasts, and LPP2 was predominantly found in cancer epithelial cells. The most upregulated gene sets in high LPP2-expressing tumors related to cell cycle progression via E2F and c-Myc pathways. Cytolytic activity scores were decreased in low LPP1-/3-expressing tumors and increased in high LPP2-expressing tumors, suggesting that this expression pattern aided in tumor immune cell evasion. This same LPP expression pattern correlated with bworsened tumor grade, increased proliferation rates, and decreased overall survival. Hence, developing therapies to either increase LPP1/3 expression in cancer cells within the tumor, or the use of LPP2 inhibitors to block endo-LPP catalytic activity, is warranted. To date, there are no specific LPP2 inhibitors with adequate bioavailability. We previously reported that tetracycline can increase LPP1/3 protein stability in breast cancer cells [24] and LPP1 expression can be induced by suppression of inflammatory signaling with dexamethasone [49]. While these drugs are most likely exerting these effects on LPP1s in a non-specific fashion, the results do provide proof-of-principle that LPP1/3 can be pharmacologically manipulated.

This study has several limitations. Although we use three independent large cohorts to validate our results, this analysis is retrospective in nature with heterogenous patient populations and treatments. Additionally, though clinical, survival, and tumor microenvironment data were derived from three independent cohorts, the various scores from Thorsson et al. [44] used to further support our findings are only available from TCGA data. While the results from bioinformatic real world data can provide useful comparators to findings from pre-clinical investigations, causative mechanisms cannot necessarily be extracted from correlative data. However, our results using human tumor genomic and clinical data are concordant with long-standing models of LPP signaling obtained from preclinical cell culture and murine model studies [8,9,15,17,19,20]. These studies demonstrate that changes in LPP mRNA expression translate directly into changes in LPP activity levels, and the mechanisms of LPP function within the cancer cell have been well demonstrated in these investigations [8,9,15,17,19,20]. Unfortunately, there are no assays available to measure specific LPP subtype enzymatic activity. Any measurement of LPP activity represents the total LPP enzyme contribution from all three enzymes, explaining why mRNA measurements have been the backbone of LPP research. Further validation of the effect of individual LPP subtype activity on cancer biology and the tumor microenvironment will require the design of selective compounds that target the individual members of the LPP family. Nevertheless, because the results in human breast cancer tumors presented in this paper are well reflected in previous translational studies on LPP biology, this work supports the therapeutic potential of decreasing the expressions of LPP2 relative to LPP1/3 in breast and possibly other cancers, and encourages the future development of novel adjunct therapies.

## 5. Conclusions

Decreased LPP1/3 and increased LPP2 expression correlate with the characteristics of an overall worse survival in breast cancer patients. Tumors with these expression profiles have higher grades, higher proliferation rates, higher rates of mutational burden, increased ability to evade the immune system, and decreased overall survival. These findings are concordant with the biology of pre-clinical models in vitro and in vivo. Therefore, we predict that this evolving understanding of LPP tumor biology should be readily translatable to the development of therapeutic interventions against aberrant expression of these enzymes, particularly inhibitors against LPP2 overexpression.

## Data Availability

All datasets are publicly available via cBioPortal (https://www.cbioportal.org (accessed on 9 October 2022)) or the Gene Expression Omnibus (GEO) repository of the United States National Institutes of Health (https://www.ncbi.nlm.nih.gov/geo (accessed on 9 October 2022)), and single-cell cohort data via the Broad Institute Single Cell Portal (https://singlecell.broadinstitute.org/single_cell (accessed on 9 October 2022)).

## Supplementary Materials

The following supporting information can be downloaded at https://www.mdpi.com/article/10.3390/cancers15082299/s1. Figure S1: LPP gene expression by breast cancer tumor nodal status and metastasis; Figure S2: Survival plots for low and high LPP gene expression in breast tumors for the estrogen receptor (ER) positive, human epidermal growth factor receptor (HER2) negative cohort for each dataset; Figure S3: Survival plots for low and high LPP gene expression in breast tumors for the human epidermal growth factor receptor (HER2) positive cohort for each dataset; Figure S4: Survival plots for low and high LPP gene expression in breast tumors for the triple negative breast cancer (TNBC) cohort for each dataset; Figure S5: Anti-cancerous immune cell correlation with LPP gene expression in breast cancer tumors; Figure S6: Pro-cancerous immune cell correlation with LPP gene expression in breast cancer tumors; Table S1: Raw GSEA output for LPP1–3.
