# Supplementary material for: Decreased Lipid Phosphate Phosphatase 1/3 and Increased Lipid Phosphate Phosphatase 2 Expression in the Human Breast Cancer Tumor Microenvironment Promotes Tumor Progression and Immune System Evasion
Source: Cancers (Basel). 2023 Apr 14;15(8):2299. doi: 10.3390/cancers15082299 (PMC10136837; doi:10.3390/cancers15082299)
Supplement: Supplementary file 1 [file cancers-15-02299-s001.zip › Supplementary Figures S1-S6.pdf]

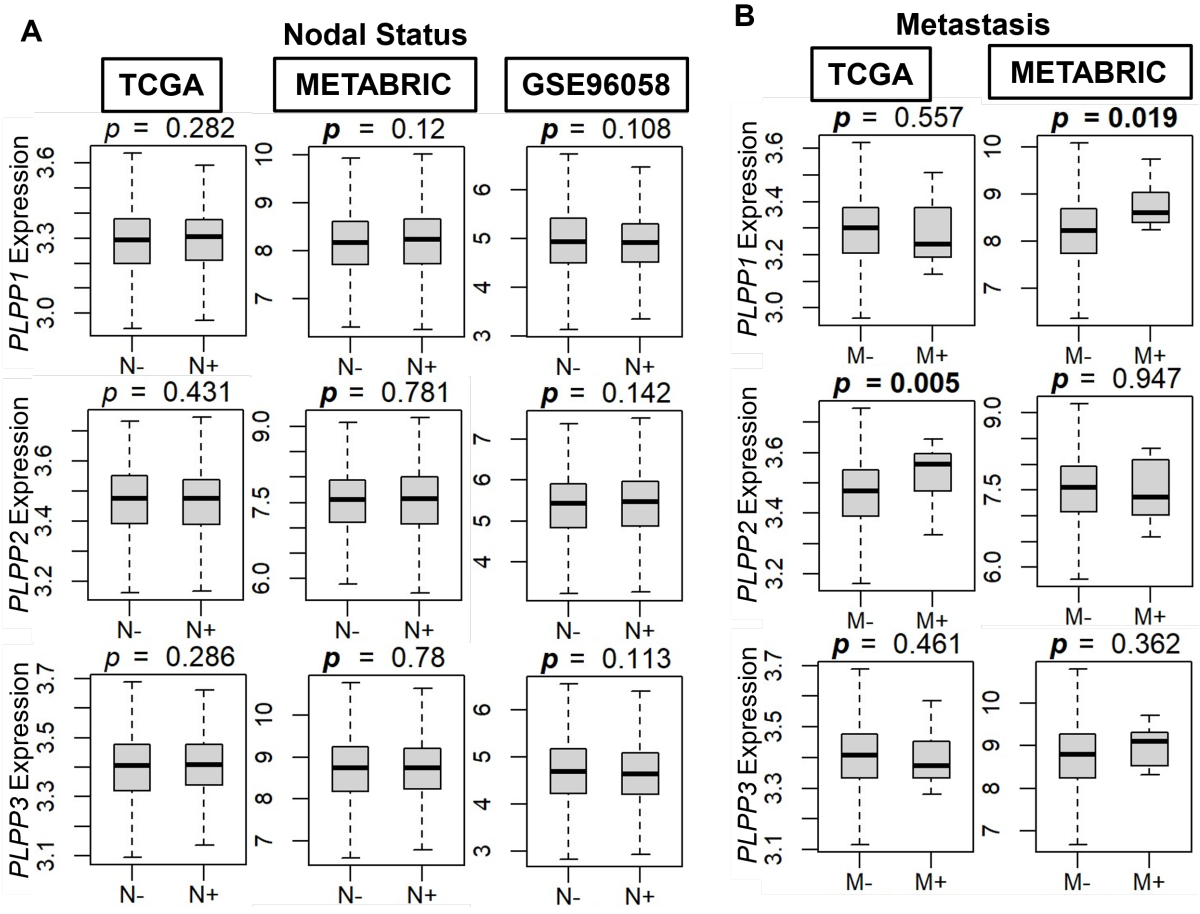

Figure S1. LPP gene expression by breast cancer tumor nodal status and metastasis. (**A**) Tumor nodal status (negative or positive). Counts by cohort: TCGA (N– (negative) n = 513, N+ (positive) n = 557), METABRIC (N– n = 993, N+ n = 911), GSE96058 (N– n = 1811, N+ n = 1162). (**B**) Tumor metastasis (negative or positive). Counts by cohort: TCGA (M– (negative) n = 1046, M+ (positive) n = 20), METABRIC (M– n = 1394, M+ n = 9). Because stage is not available for the GSE96058 cohort, metastasis data is also unavailable for this cohort. The bolded center bar within the box plots represents the median; the lower and upper box bounds represent the 25th and 75th percentiles, respectively; and the lower and upper tails represent the minimum and maximum values, respectively.

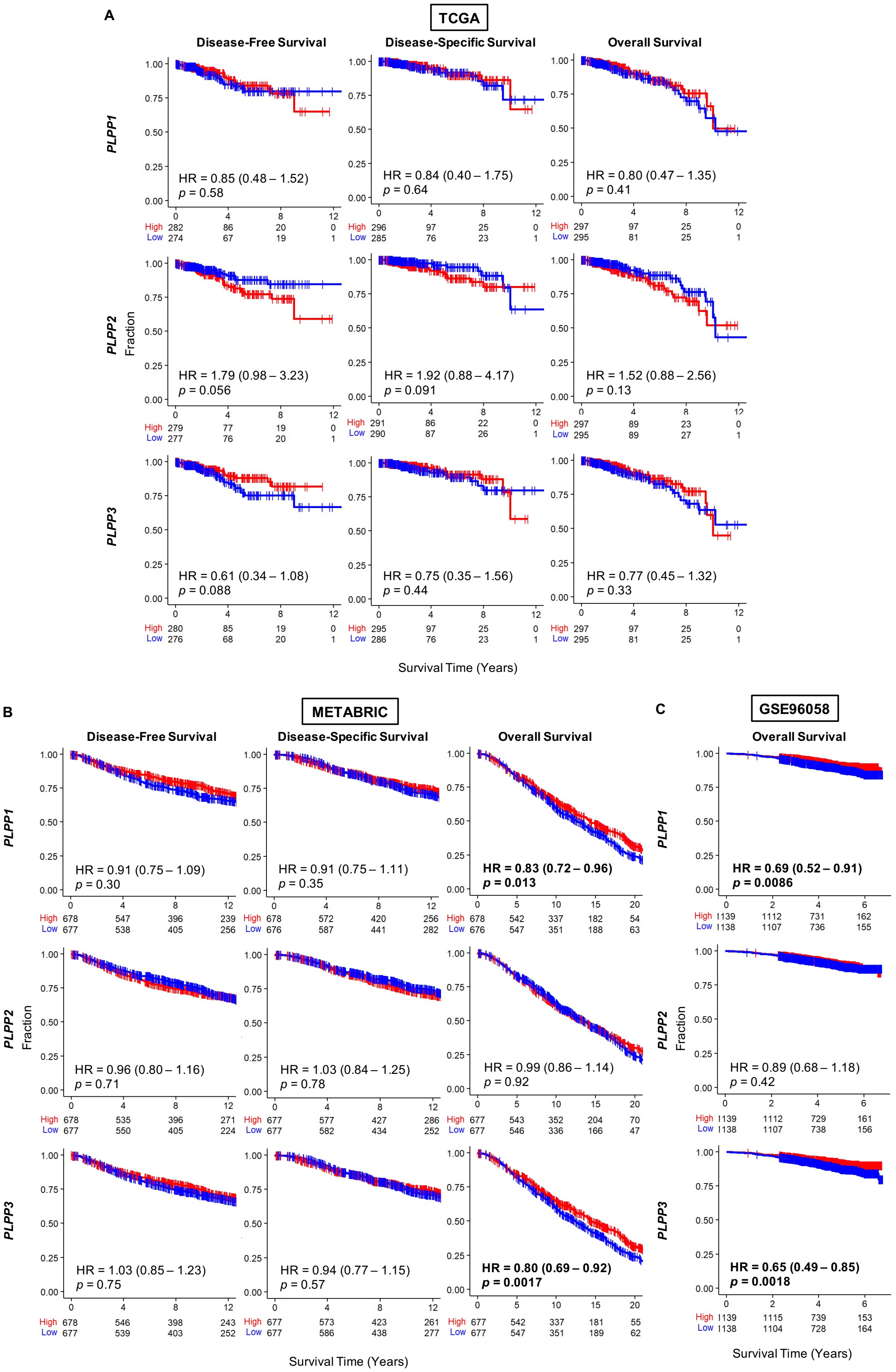

Figure S2. Survival plots for low and high LPP gene expression in breast tumors for the estrogen receptor (ER) positive, human epidermal growth factor receptor (HER2) negative cohort for each dataset. (**A**) TCGA cohort results. (**B**) METABRIC cohort results. (**C**) GSE96058 cohort results. Patients at risk for each time point are listed along the x-axis. LPP expression is dichotomized into low and high groups by the median. The hazard ratio (HR) compares the high group against the low group.

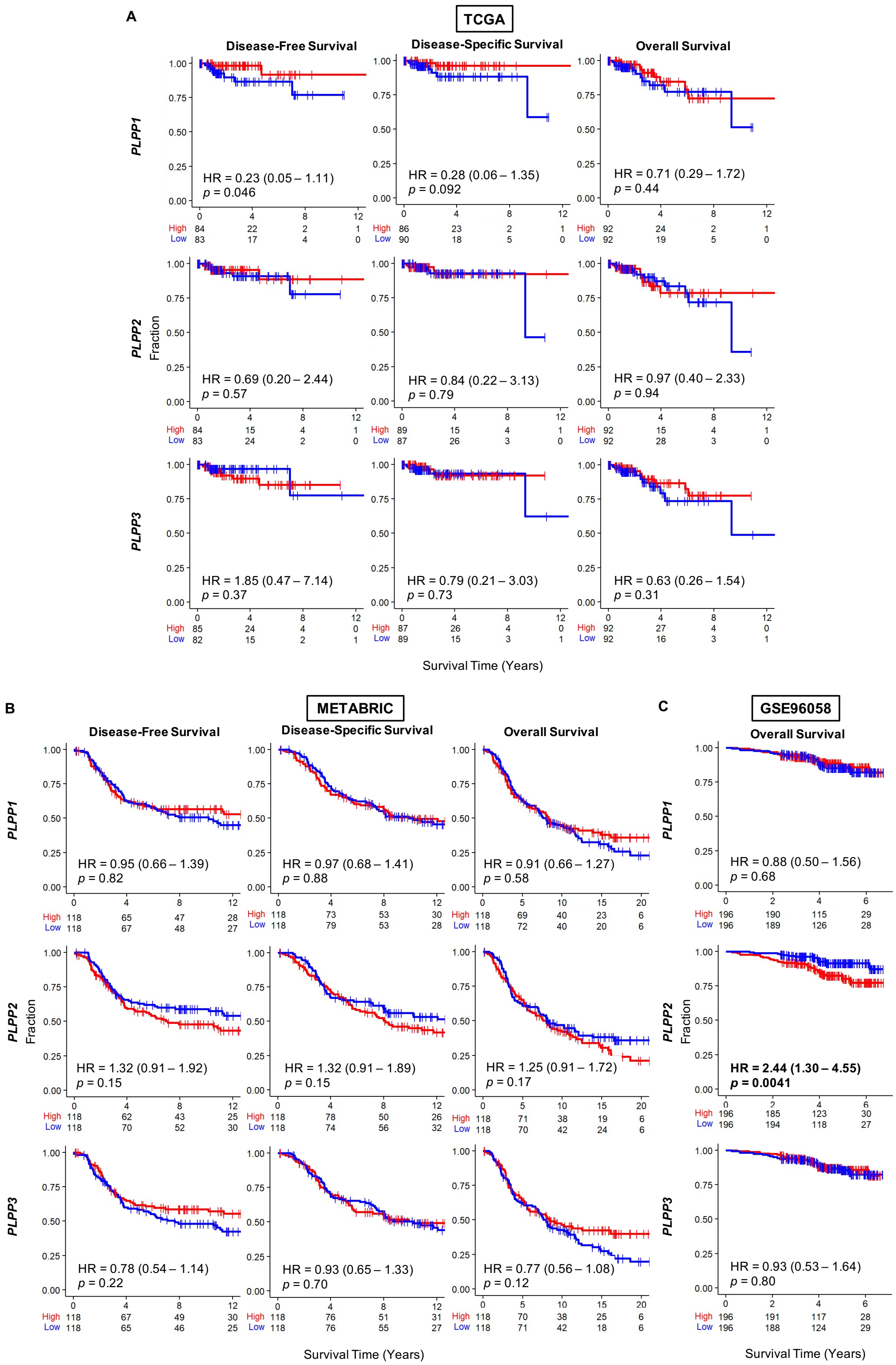

Figure S3. Survival plots for low and high LPP gene expression in breast tumors for the human epidermal growth factor receptor (HER2) positive cohort for each dataset. (**A**) TCGA cohort results. (**B**) METABRIC cohort results. (**C**) GSE96058 cohort results. Patients at risk for each time point are listed along the x-axis. LPP expression is dichotomized into low and high groups by the median. The hazard ratio (HR) compares the high group against the low group.

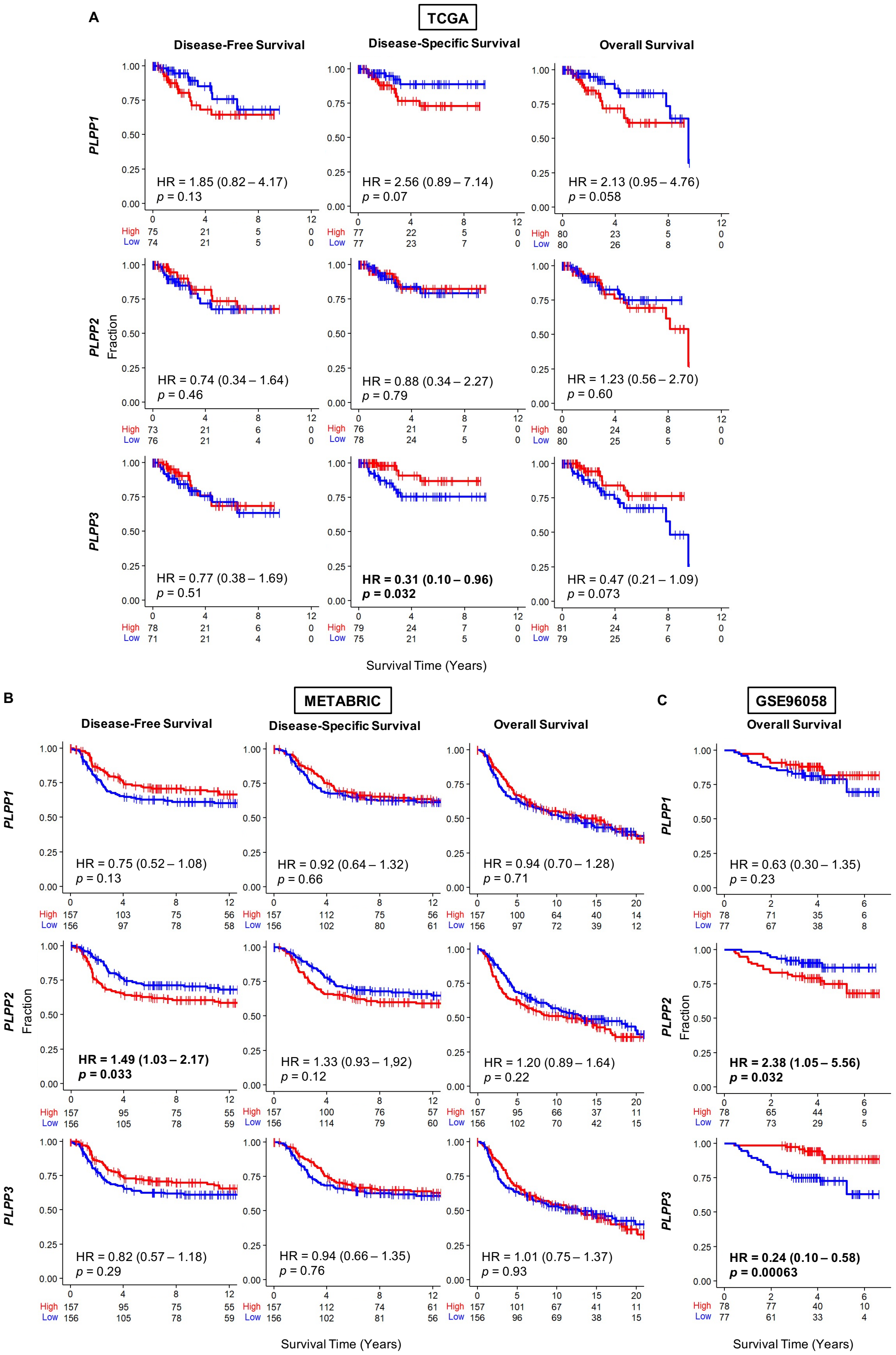

Figure S4. Survival plots for low and high LPP gene expression in breast tumors for the triple negative breast cancer (TNBC) cohort for each dataset. (**A**) TCGA cohort results. (**B**) METABRIC cohort results. (**C**) GSE96058 cohort results. Patients at risk for each time point are listed along the x-axis. LPP expression is dichotomized into low and high groups by the median. The hazard ratio (HR) compares the high group against the low group.

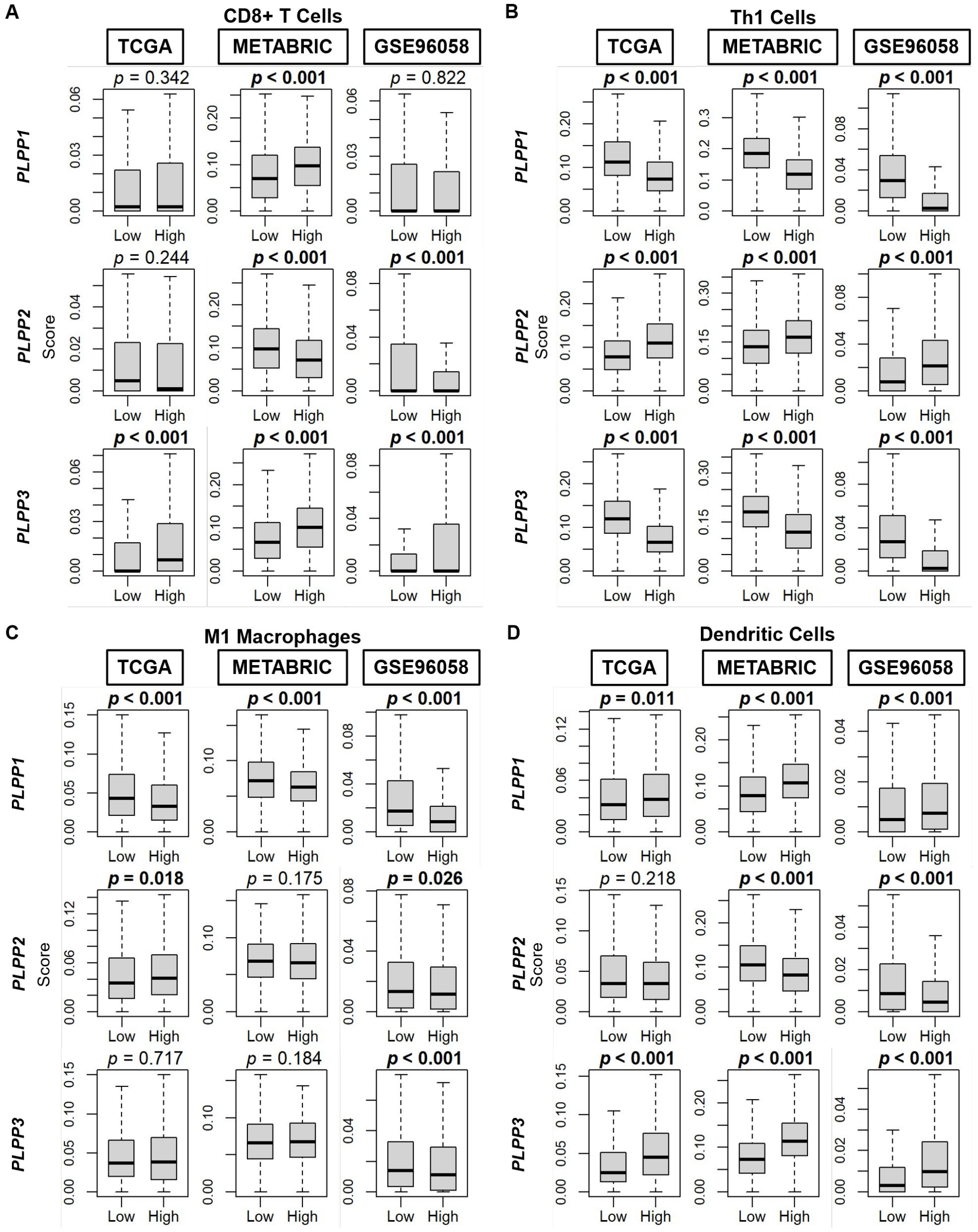

Figure S5. Anti-cancerous immune cell correlation with LPP gene expression in breast cancer tumors. (**A**) Box plots of CD8+ T cell composition. (**B**) Box plots of T–helper 1 (Th1) cell composition. (**C**) Box plots of M1 macrophage composition. (**D**) Box plots of dendritic cell composition. All data based on the xCell algorithm for the TCGA, METABRIC, and GSE96058 cohorts. LPP gene expression is dichotomized into low and high groups by the median. The bolded center bar represents the median; the lower and upper box bounds represent the 25th and 75th percentiles, respectively; and the lower and upper tails represent the minimum and maximum values, respectively.

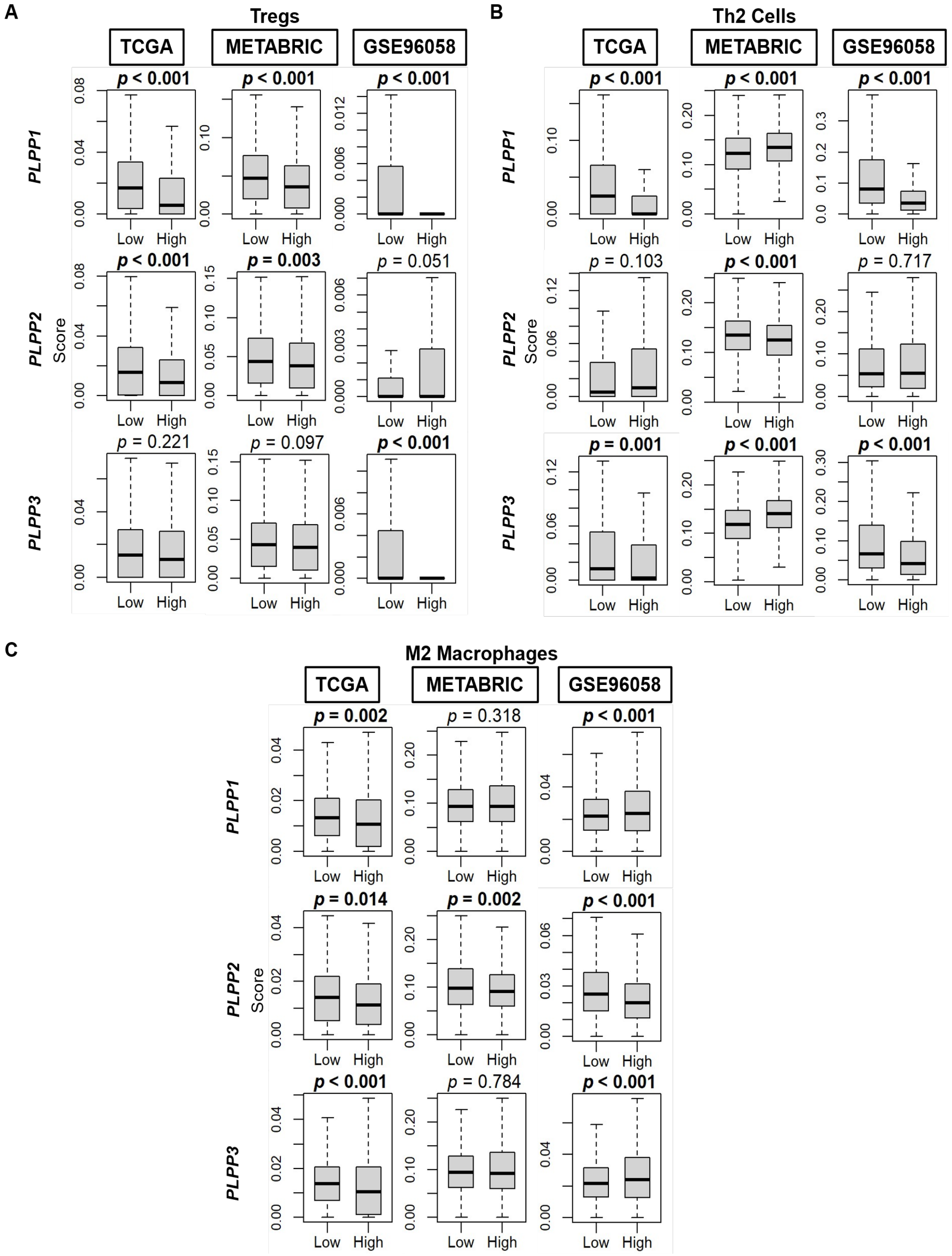

Figure S6. Pro-cancerous immune cell correlation with LPP gene expression in breast cancer tumors. (**A**) Box plots of T regulatory (Treg) cell composition. (**B**) Box plots of T–helper 2 (Th2) cell composition. (**C**) Box plots of M2 macrophage composition. All data based on the xCell algorithm for the TCGA, METABRIC, and GSE96058 cohorts. LPP gene expression is dichotomized into low and high groups by the median. The bolded center bar represents the median; the lower and upper box bounds represent the 25th and 75th percentiles, respectively; and the lower and upper tails represent the minimum and maximum values, respectively.
